# New Genetic Biomarkers of the Overlap Syndrome Tension-Type Headache and Arterial Hypertension

**DOI:** 10.3390/genes13101823

**Published:** 2022-10-09

**Authors:** Polina V. Alyabyeva, Olga V. Chastina, Marina M. Petrova, Natalia V. Lareva, Natalia P. Garganeeva, Galina A. Chumakova, Marina S. Cherniaeva, Natalia A. Shnayder

**Affiliations:** 1 Shared Core Facilities Molecular and Cell Technologies, V.F. Voino-Yasenetsky Krasnoyarsk State Medical University, 660022 Krasnoyarsk, Russia; 2 Department of Therapy of Faculty of Postgraduate Education, Chita State Medical Academy, 672000 Chita, Russia; 3 Department of General Medical Practice and Outpatient Therapy, Siberian State Medical University, 634050 Tomsk, Russia; 4 Department of Therapy and General Medical Practice with a Course of Additional Professional Education, Altai State Medical University, 656038 Barnaul, Russia; 5 Department of Internal and Preventive Medicine, Central State Medical Academy of the Presidential Administration, 121359 Moscow, Russia; 6 Institute of Personalized Psychiatry and Neurology, V.M. Bekhterev National Medical Research Center for Psychiatry and Neurology, 192019 Saint Petersburg, Russia

**Keywords:** tension-type headache, arterial hypertension, comorbidity, overlap syndrome, nitric oxide, genetic biomarker, *NOS1*, rs3782218

## Abstract

Background: Nitric oxide (NO) is an important autocrine and paracrine signaling molecule that plays a crucial role in cardiovascular physiology and pathology regulation. NO is an important molecule involved in regulation of cerebral and extra cerebral cranial blood flow and arterial diameters. Reduced bioavailability of NO in the endothelium is an important precursor for impaired vasodilation and arterial hypertension (AH). Furthermore, NO is involved in nociceptive processing. A NO-induced biphasic response with immediate and a delayed headache is typical for chronic tension-type headaches (TTH) in humans. The aim was to study the association of allelic variants and genotypes of the single nucleotide variant (SNV) rs3782218 of the *NOS1* gene with the TTH and AH overlap syndrome development in middle age adults. Materials and Methods: We observed 91 Caucasian participants who resided in Krasnoyarsk city: group 1 (TTH and AH overlap syndrome)—30 patients; group 2 (AH without headache)—30 patients; group 3 (control)—31 healthy volunteers. The diagnosis of AH was based on criteria of the European Society of Cardiology and the European Society of Hypertension (2018) и criteria of the Russian Society of Cardiology (2020). Diagnosis of TTH was based on criteria of the International Classification of Headache Disorders (2018). Real-time polymerase chain reaction was used for the determination of allelic variants and genotypes of the SNV rs3782218 of the *NOS1* gene in all groups of participants. Results: The frequency of the minor allele T of rs3782218 was statistically significantly higher by 16.7 times in group 1 (TTH and AH) compared to group 3 (control): 26.7% versus 1.6%, respectively (*p*-value = 0.000065) and 3.2 times higher in group 1 (TTH and AH) compared to group 2 (AH without headache): 26.7% versus 8.3%, respectively (*p*-value = 0.008). The frequency of the heterozygous (CT) genotype was statistically significantly higher in group 1 (TTH and AH) compared to group 3 (control): 40.0% versus 3.2% (*p*-value = 0.000454) and in group 1 (TTH and AH) compared to group 2 (AH without headache): 40.0% versus 16.7% (*p*-value = 0.045). The minor allele T was statistically significantly associated with a high risk of developing the TTH and AH overlap syndrome compared with the controls (odds ratio (OR) = 22.2 (95% confidential interval (CI): 2.8–173.5)) and compared with AH without headache (OR = 4.0 (95% CI: 1.4–11.8)). Although the frequency of the minor allele T was 5.2 times higher in group 2 (AH without headache) compared with group 3 (control), there were not statistically significantly differences (*p*-value = 0.086). Conclusion: Thus, the minor allele T of rs3782218 of the *NOS1* gene is an important genetic biomarker for a high risk of developing the TTH and AH overlap syndrome in hypertensive patients.

## 1. Introduction

The tension-type headache (TTH) is the most common type of primary headache, and arterial hypertension (AH) is a prevalent condition worldwide. Many studies support the hypothesis that TTH patients have a higher risk of developing AH, while hypertensive patients have a higher risk of developing TTH than in the general population. The relationship between TTH and AH is potentially of great pathophysiological and clinical interest as an overlap syndrome, but it is poorly understood [1]. This allows us to hypothesize the presence of the TTH and AH overlap syndrome [2].

Nitric oxide (NO) is an important autocrine and paracrine signaling molecule that plays a crucial role in cardiovascular and cerebrovascular disorders [3]. Reduced bioavailability of NO in the vascular endothelium is an important predictor for impaired vasodilation and AH. Furthermore, NO is involved in nociceptive processing. A NO-induced biphasic response with immediate and a delayed headache is typical for chronic TTH in humans [2].

As we know, NO-synthases (NOSs) are expressed in three isoforms: neuronal (nNOS), inducible (iNOS), and endothelial (eNOS) [4]. The functional activity of these isoenzymes depends on the carriage of wild, highly functional, low functional, and non-functional alleles of single nucleotide variants (SNVs) of *NOS1*, *NOS2*, and *NOS3* genes encoding the enzyme isoforms [5].

Genetic and physiological mechanisms of TTH and AH have not yet been clarified. Studies in which TTH was induced by intravenous infusions of glyceryl trinitrate (an exogenous NO donor) and histamine (which liberates NO from vascular endothelium) suggest that NO is likely to be a responsible molecule. The release of NO from blood vessels, perivascular nerve endings or from central nervous system tissue, is an important molecular trigger in headache [6]. Sarchielli P. et al. (2001) assessed the variations in L-arginine/NO pathway activity and platelet cyclic guanosine 3,5-monophosphate (cGMP) levels in patients affected by chronic TTH. A reduction in platelet aggregation response was found, it was coupled with increased NO and cGMP production. A significant increase in cytosolic Ca(2+) concentration was also detected compared to the control individuals. This was accompanied by a reduced platelet content and collagen-induced secretion of serotonin and increased content of NO in patients with TTH. The increased NOS activity shown in platelets of TTH patients reflects an analogous central up-regulation of NOS activity in the spinal horn/trigeminal nucleus and supraspinal structures; these structures are involved in the modulation of nociceptive input from myofascial cranial structures contributing to central sensitization [7]. Chronic TTH may be caused by a prolonged painful input from pericranial myofacial tissues, such as tender points, which results in central sensitization (increased excitability of neurons in the central nervous system. Animal studies have shown that sensitization of pain pathways may be caused by or associated with the activation of nNOS and generation of NO. Furthermore, it has been shown that NOS inhibitors reduce central sensitization in animal models of persistent pain [8]. nNOS is involved in the induction but not the maintenance of nerve growth factor (NGF) caused by facilitation of nociception in the brainstem [9]. The results from an experimental animal model may support the idea of nNOS as potential target for pharmacological treatment of TTH [7]. The role of nNOS in central sensitization induced by an intradermal capsaicin injection was investigated by Wu J. et al. (2001) [10].

As known, NO is the main mediator of endothelial dysfunction underlying the development of AH [11]. Kelm M. et al. (1996) identified three causes of NO-dependent vasospasm (impaired NO-dependent vasodilation) in patients with AH: (1) reduced NO synthesis by endothelial cells due to impaired signal transduction and/or decreased NO synthase activity, (2) accelerated NO degradation in the vessel wall, and (3) structural disorders in the vessel wall leading to a general decrease in the dilator capacity of resistant arteries [12]. Increased NO biosynthesis enhances angiogenesis. Conversely, angiogenesis is impaired when NO levels are reduced. That is, conditionally, angiogenesis begins in response to hypoxia. Thus, signs of impaired angiogenesis and, as a result, microvascular rarefaction, are revealed in AH [13]. It is important that the degree of AH does not affect angiogenesis, and microvascular rarefaction is also observed in normotensive people with a family history of hypertension. The genetic basis of these mechanisms has also been studied. Recent studies have confirmed that the pathway of L-arginine conversion to NO is impaired not only in people with AH, but also in people with normotension with a history of essential AH [14].

nNOS may be important molecular predictor of developing of TTH and AH overlap syndrome, because it is mainly expressed in the brain, but has in addition to its presence in cardiomyocytes also been identified in vascular smooth muscle and skeletal muscle. The nNOS is coding by *NOS1* gene which localized on chromosome 12q24 [15].

The genetic predisposition to TTH and TTH and AH overlap syndrome is poorly understood [2] compared to other similar overlap syndromes, for example, migraine and AH overlap syndrome [16]. However, according to epidemiological studies, the prevalence of TTH among hypertensive patients ranged from 33.4% to 85.0% [17,18], mean—59.2%, and the prevalence of migraine among hypertensive patients ranged from 15.0% to 66.6% [17,18], mean—40.8%. In general, the prevalence of TTH among patients with AH (TTH and AH overlap syndrome) was 1.45 times higher compared with the prevalence of migraine among patients with AH (migraine and AH overlap syndrome) [1].

So, in recent years, isolated associative genetic studies have appeared that make it possible to find the key to understanding the genetic predisposition for impaired NO synthesis in patients with TTH and AH overlap syndrome. However, the results of these studies are contradictory [2] and there is no consensus on this issue.

Of greatest interest to researchers are two single nucleotide variants (SNVs) of *NOS1* gene, which may be genetic predictors of the TTH and AH overlap syndrome development in Caucasians, including rs3782218 (117771511 C>T) and rs7314935 (117718837 G>A) [19]. The rs3782218 is the most promising.

The aim is the study of association of allelic variants and genotypes of the SNV rs3782218 of the *NOS1* gene with the TTH and AH overlap syndrome development in middle age adults.

## 2. Materials and Methods

### 2.1. Data Collection

The study was a part of a comprehensive study on the topic “Clinical and genetic predictors of the tension-type headache and arterial hypertension overlap syndrome”, registration No. 122030300108-6 dated 3 March 2022.

The study was approved by the local ethics committee of the V.F. Voino-Yasenetsky Krasnoyarsk State Medical University (KrasSMU), protocol No. 101/2020 dated 31 October 2020. The study was carried out in accordance with the requirements of the World Medical Association Declaration of Helsinki—ethical principles for medical research involving human subjects, declared at the 64th General Assembly of the World Medical Associations (Fortaleza, Brazil, 2013) [20]. All patients and healthy volunteers signed a voluntary informed consent to participate in this study. Patients and healthy volunteers were not rewarded for participating in the study.

The study was supported by the intra-university grant to support the research of young scientists of the KrasSMU (No. 462-base dated 12 July 2021).

The study was carried out on the basis of Shared Core Facilities Molecular and Cell Technologies include the Department of General Medical Practice, the Department of Outpatient’s Therapy and Family Medicine, the Department of Medical Genetics and Clinical Neurophysiology of the KrasSMU within scientific cooperation with the Institute of Personalized Psychiatry and Neurology of V.M. Bekhterev National Medical Research Center for Psychiatry and Neurology, and medical universities of Siberia (Chita State Medical Academy, Altai State Medical University, Siberian State Medical University) in the period from October 2020 to July 2022.

The research was an open, observational, cross-sectional, and case–control design.

### 2.2. Study Population

The size of the sample was calculated using Altman’s nomogram method [21] using the online calculator MedStatistic [22]. A significance level (alpha) of 5% (0.05) and power (1-beta) of 90% (0.9) was used. The value of the expected frequency of the phenomenon in the main group (development of TTH in patients with AH, i.e., TTH and AH overlap syndrome) was 59.2% (taking into account our previous analysis [1]). The value of the expected frequency of the phenomenon in the control group (no headache in patients with AH) was 15.0% (also taking into account our previous analysis [1]). The value of the standardized difference was equal to 0.91. So, according to the Altman’s nomogram, the number of studied cases should be at least 26 in each group. The dropout rate for this study was chosen as 10%. Thus, the sample size of each group had to be at least 29 participants each, and it is big enough to detect the differences between groups.

The measures used to control sampling bias were as follows: determined the target population and the sampling frame (the list of persons who were included in the sample); to the extent possible, match the sampling frame with the target population to reduce the risk of sampling bias; made surveys of study participants as short and as accessible as possible; convenience samples were avoided.

We observed 91 Caucasian participants who resided in Krasnoyarsk city: group 1 (TTH and AH overlap syndrome)—30 patients (mean—52.7 ± 5.7 years); group 2 (AH without headache)—30 patients (mean—53.6 ± 7.1 years); group 3 (control)—31 healthy volunteers (mean—52.7 ± 6.7 years). The diagnosis of AH was based on the criteria of the European Society of Cardiology and the European Society of Hypertension (2018) [23] and criteria of the Russian Society of Cardiology (2020) [24]. Diagnosis of TTH was based on the criteria of the International Classification of Headache Disorders (2018) [25].

The criteria for inclusion in group 1 (TTH and AH overlap syndrome) were as follows:

1. Russian-speaking residents of a large industrial city of the Siberian Federal District of the Russian Federation (Krasnoyarsk);
2. Caucasians;
3. Adults: World Health Organization (WHO) [26] first median age (male: 45 to 65 years old, female: 40 to 60 years old);
4. AH (diagnosed by a cardiologist or general practitioner);
5. TTH (diagnosed by a neurologist or general practitioner);
6. Signed voluntary informed consent to participate in this study.
7. Criteria for inclusion in group 2 (AH without headache):
8. Russian-speaking residents of a large industrial city of the Siberian Federal District of the Russian Federation (Krasnoyarsk);
9. Caucasians;
10. Adults: WHO [26] first median age (male: 45 to 65 years old, female: 40 to 60 years old);
11. AH (diagnosed by a cardiologist or general practitioner);
12. Signed voluntary informed consent to participate in this study.

The criteria for inclusion in group 3 (control) were as follows:

1. Russian-speaking residents of a large industrial city of the Siberian Federal District of the Russian Federation (Krasnoyarsk);
2. Caucasians;
3. Adults: WHO [26] first median age (male: 45 to 65 years old, female: 40 to 60 years old);
4. Signed voluntary informed consent to participate in this study.

The criteria for exclusion from the study were as follows:

1. Non-Russian-speaking residents of a large industrial city in the Siberian Federal District of the Russian Federation (Krasnoyarsk): migrants, representatives of small ethnic groups in Siberia;
2. Asians;
3. Refusal to participate in this study;
4. Refusal to comply with the full protocol of this study;
5. Participation in other studies;
6. Severe cognitive disorders and dementia of any etiology;
7. Acute infectious diseases;
8. Diabetes;
9. Other primary headaches (except TTH);
10. Secondary headaches;
11. Traumatic brain injury;
12. History of stroke and transient ischemic attacks;
13. Epilepsy and epileptic syndromes;
14. Chronic renal and hepatic insufficiency;
15. Chronic heart failure (New York Heart Association Functional Classification: class II and above).

### 2.3. Genetic Analysis

Blood samples were drawn by vein-puncture after an overnight fast of 12 h. The samples were separated by centrifugation, and aliquots were stored at −86 °C until analyses. Genomic DNA was extracted following a standard protocol [27]. Primers were designed according to the Applied Biosystems protocol (USA).

The PCR reaction consisted of the following components: 2.5x reaction mix for real-time PCR, which included (a pair of allele-specific TaqMan MGB probes (comprising a DNA minor groove binding (MGB) fragment at the 3’ end and an integrated NFQ quencher); deoxynucleoside triphosphates; PCR buffer; MgCl2), Taq DNA polymerase with enzyme-inhibiting antibodies, ultra-pure water. For DNA elution, buffers were added to the tubes, resuspended, and incubated in a thermostat for 5 min at 65 °C, periodically vortexing. To remove the sorbent freed from DNA, the tubes were centrifuged for 1 min at a rotation speed of 12,000 rpm. The supernatant containing DNA was transferred into clean labeled tubes and stored at −20 °C.

The allelic variants and genotypes of the SNV rs3782218 of the NOS1 gene on chromosome 12q24 was determined in all groups of participants by real-time polymerase chain reaction (RT-PCR) using diagnostic equipment Rotor-Gene 6000 (Corbett Life Science, Sydney, Australia) and technology allelic discrimination of TaqMan and fluorescent probes (Applied Biosystems, Waltham, MA, USA).

For rs3782218, C is the major allele and T is the minor allele. The following designations were adopted to denote variants of the rs3782218 genotypes: homozygous high-producing genotype—CC (cytosine/cytosine), heterozygous genotype—CT (cytosine/thymine), and homozygous genotype for the low-producing allele—TT (thymine/thymine).

### 2.4. Statistical Analysis

All data were analyzed using the ISB SPSS 22.0 (SPSS Inc., Chicago, IL, USA) program.

Deviations from the Hardy–Weinberg Equilibrium (HWE) expectations were tested by the chi-square test. The gene counting method tested the genotype distribution and allele frequencies. The chi-square test was applied to evaluate differences in genotype distributions and allele frequencies between groups. The risk of developing the TTH and AH overlap syndrome and AH was assessed using the odds ratio (OR, 95% confidence interval (CI)). *p*-values < 0.05 were considered statistically significant.

## 3. Results

### 3.1. Baseline Clinical Characteristic

**Figure 1:**
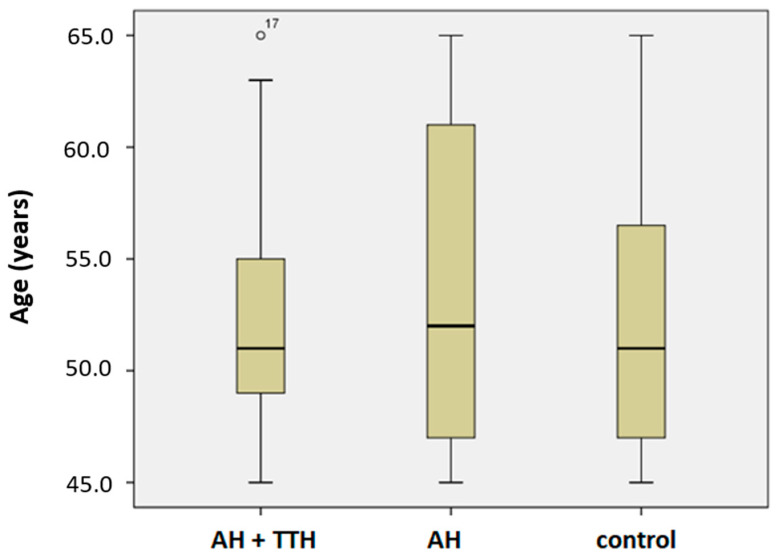
Characteristics of groups by age. Note: AH—arterial hypertension; TTH—tension-type headache.

The groups were comparable in terms of age (*p*-value > 0.05, Figure 1) and gender (*p*-value > 0.05).

Relevant baseline clinical characteristics and traditional AH risk factors of the participants are shown in Table 1. There were no statistically significant differences in duration of AH anamnesis (*p*-value > 0.05) and age at onset of hypertension (*p*-value > 0.05). Significant differences were observed in alcohol consumption (*p*-value < 0.05), smoker status (*p*-value < 0.05), salt intake (*p*-value < 0.05), and level of physical activity (*p*-value < 0.05) in groups 1 (TTH and AH), and 2 (AH without headache) compared with group 3 (control).

### 3.2. Frequency Distribution of Genotypes and Alleles in the TTH and AH/AH Patients and Control

In the study on the carriage of rs3782218 in the NOS1 gene, the data obtained were found to be consistent with the HWE law in group 1 (TTH and AH overlap syndrome)*—*χ^2^ = 0.016; *p*-value = 0.99, group 2 (AH without headache)*—*χ^2^ = 0.248; *p*-value = 0.88 and group 3 (control)*—*χ^2^ = 0.008; *p*-value = 0.1. Thus, to evaluate the results obtained, we used two models: multiplicative (to estimate the frequency of alleles) and additive (to estimate the frequency of genotypes).

The frequency of the major allele C of rs3782218 of the *NOS1* gene was statistically significantly lower in group 1 (TTH and AH) compared to group 3 (control): 73.3% versus 98.4%, respectively (*p*-value = 0.000065) and the frequency of the minor allele T of rs3782218 was statistically significantly higher (16.7 times) in group 1 (TTH and AH) compared to group 3 (control): 26.7% versus 1.6%, respectively (*p*-value = 0.000065); Table 2. The frequency of heterozygous (CT) genotype was statistically significantly higher in group 1 (TTH and AH) compared to group 3 (control): 40.0% versus 3.2% (*p*-value = 0.000454); Table 2.

The minor allele T was statistically significantly associated with a high risk of developing the TTH and AH overlap syndrome compared with the control (OR = 22.2 (95% CI: 2.8—173.5)); Table 3.

The frequency of the major allele C of rs3782218 of the *NOS1* gene was statistically significantly lower in group 1 (TTH and AH) compared to group 2 (AH without headache): 73.3% versus 91.7%, respectively (*p*-value = 0.008) and the frequency of the minor allele T of rs3782218 was statistically significantly higher (3.2 times) in group 1 (TTH and AH) compared to group 2 (AH without headache): 26.7% versus 8.3%, respectively (*p*-value = 0.008); Table 2. The frequency of heterozygous (CT) genotype was statistically significantly higher in group 1 (TTH and AH) compared to group 2 (AH without headache): 40.0% versus 16.7% (*p*-value = 0.045); Table 2.

The minor allele T was statistically significantly associated with a high risk of developing the TTH and AH overlap syndrome compared with AH without headache (OR = 4.0 (95% CI: 1.4–11.8)); Table 3.

However, we did not find statistically significant differences in the frequency of the major allele C in group 2 (AH without headache) versus group 3 (control): 91.7% versus 98.4%, respectively (*p*-value = 0.086). Although, the frequency of the minor allele T was 5.2 times higher in group 2 (AH without headache) compared with group 3 (control), but there were not statistically significantly differences (*p*-value = 0.086); Table 2.

## 4. Discussion

**Figure 2:**
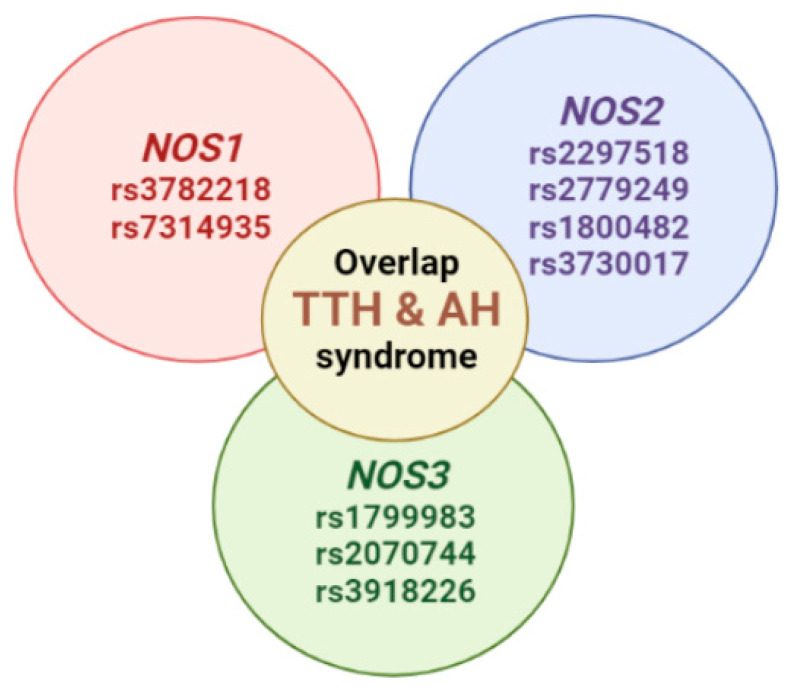
Potential SNVs of *NOS1, NOS2,* and *NOS3* genes predisposed to the tension-type headache and arterial hypertension overlap syndrome (modification by authors from [2]).

Patients with overlap syndromes have features of more than one defined disease. Overlap syndromes are therefore highly heterogeneous as many combinations of clinical and genetic features can occur [28]. An overlap syndrome is a medical condition which shares features of at least two more widely recognized disorders. This syndrome usually present subacute with clinical manifestations that can include different organ systems. Our earlier review demonstrated the potential role of NOS in the development TTH and AH overlap syndrome (Figure 2).

The prognostic role of the *NOS1* gene in the development of the common TTH and AH overlap syndrome has not been studied. Our study testifies to the importance of planning and conducting associative genetic research in the role of the *NOS1* gene as a new genetic biomarker of the TTH and AH overlap syndrome in various racial and ethnic groups.

**Figure 3:**
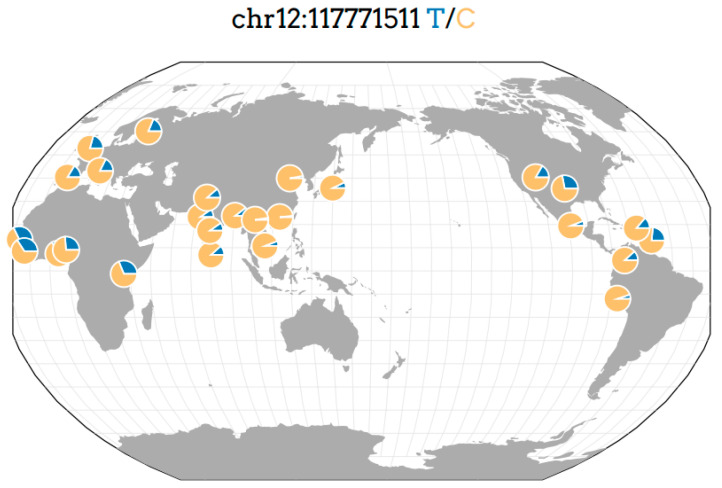
Geography of genetic variant rs3782218 of the *NOS1* gene in the world [29].

This is the first report on the association of rs3782218 of the *NOS1* gene on chromosome *12q24* with TTH and AH overlap syndrome in European population from Siberia (Russia) (Figure 3 [29]).

Ninety one (91) participants were successfully genotyped for rs3782218 (117771511 C>T). To our knowledge, this is the first publication describing this SNV as a prognostic genetic biomarker of the TTH and AH overlap syndrome [2].

However, the association of the primary headache with SNVs of the *NOS1* gene was studied, but with other SNVs and in patients with migraines [16]. No associations were found in patients with migraines in Turkish (Alaşehirli B. et al. (2012) [30]), Japanese (Ishii M. et al. (2014) [31]), and Spanish (García-Martín E. et al. (2015) [32]) populations. To our knowledge, the role of this SNV in TTH development has not been previously investigated, either in the general population or in hypertensive patients.

It also has previously been investigated as a genetic biomarker for AH. So, Levinsson A. et al. (2014) found that the carriage of the T allele rs3782218 of the *NOS1* gene reduces the risk of developing AH (OR = 0.81, 95% CI = 0.67–0.97, *p*-value = 0.02) in the Swedish population [19]. While our study showed that the T allele rs3782218 of the *NOS1* gene did not statistically significantly affect the risk of developing AH (OR = 5.55, 95% CI = 0.63–48.95, *p*-value = 0.086), however, the T allele rs3782218 of the *NOS1* gene increases the risk of developing the TTH and AH overlap syndrome (OR = 22.18, 95% CI = 2.84–173.54, *p*-value = 0.000065) in the European Siberian population of Russia. In our study, the heterozygous (CT) genotype was significantly associated with the TTH and AH overlap syndrome (OR = 20.0, 95% CI = 2.4–166.97, *p*-value = 0.000419).

Thus, our data supplement our previous studies of the role of the SNV-marker rs3782218 of the *NOS1* gene in the Russian population of Eastern Siberia, but do not agree with the results of Levinson’s (2014) [19] study performed in the Swedish population (Northern Europe). It should be noted that the sample size and the absence of other ethnic control groups (for example, Asians living in Eastern Siberia), which could supplement the existing picture of the allele distribution, do not allow for general conclusions to be drawn at this stage regarding the strength of the association of this marker with a genetic predisposition to TTH and AH overlap syndrome in Eastern Siberia.

Nevertheless, the obtained data, by way of dozens of samples, determine the feasibility of further studies, while it is necessary to take into account the difference in the distribution of genetic biomarkers in different populations (population stratification factor). Thus, the frequency of allelic variants of SNV rs3782218 of the *NOS1* gene, differently presented in the population of the European and Asian regions (Figure 3 [21]), may differ both within different populations of Western and Eastern Europe, and within the population system of Russia. According to the available data, the gene pool of the studied population living in Eastern Siberia is less affected by the “gene influx” from the countries of Western Europe, and, on the contrary, more affected by the “gene influx” from the countries of the Asian region, according to the central and European parts of Russia. This has been demonstrated in associated genetic studies of other neurological diseases [33].

So, the studied SNV-marker rs3782218 of the *NOS1* gene can be useful for predicting the risk of TTH and AH overlap syndrome in the consideration area. At the same time, the risk of this syndrome developing increases with the heterozygous (CT) genotype. It is possible that an increase in the sample size in further population studies may provide new information on the frequency of the rare homozygous (TT) genotype in Caucasians of Eastern Siberia.

This is important from a scientific and clinical point of view, because a new class of drugs that inhibit nNOS has been proposed in recent years, both for the treatment of TTH and AH. A new strategy for predicting and disease-modifying therapy of the common TTH and AH overlap syndrome can increase the effectiveness and safety of treatment, improve patient quality of life, and reduce the risk of life-threatening cardiovascular complications.

## 5. Limitations

A limitation of our study is the impossibility of constructing a recessive model (TT/CC + CT), since the homozygous TT genotype was not found among participants of group 2 (AH without hypertension) and group 3 (control) in the study population (the European Siberian population of Russia).

Despite power analysis showing that the sample size of each group is big enough to detect the differences between groups, it is still a relatively small sample size, since the homozygous TT genotype was not found among participants of group 2 (AH without headache) and group 3 (control) in the study population. Therefore, the relatively small sample size should be acknowledged as a possible limitation of the study. It is unclear if this apparent low diversity is real or due to sampling bias. However, we are now aware of a possible sampling bias and should take it into account when continuing our study in the future.

Another limitation of our study is the absence of a group of TTH patients without AH. This is explained by the fact that the main idea of this study was to search for new genetic biomarkers of a clinically unfavorable TTH and AH overlap syndrome in hypertensive patients. However, our study is ongoing, which will allow us to present new results in the future.

The additional limitation of the study is that possible confounding factors have not been included in the statistical analyses.

## Figures and Tables

**Table 1:** General characteristics of the cases (groups 1 and 2) and control (group 3).

General Characteristics	TTH and AH	AH	Control	*p*-Value
Duration of AH anamnesis (mean)	12.4 ± 12.4	15.8 ± 9.9	-	0.060
Age at onset of AH (mean)	40.6 ± 11.7	37.8 ± 11.5	-	0.216
Non-alcohol (%)	26.7	26.7	22.6	0.914
Rare alcohol (%)	66.7	60.0	77.4	0.111
Active alcohol (%)	6.6	13.3	0.0	0.048
Non-smokers (%)	46.6	56.7	70.9	0.047
Active smokers (%)	36.7	30.0	19.3	0.319
Low-salt diet (%)	16.6	23.3	22.6	0.784
High-salt diet (%)	26.8	10.0	3.2	0.021
Low physical activity (%)	43.3	20.0	16.1	0.034
Moderate physical activity (%)	46.7	53.3	51.6	0.866
Intense physical activity (%)	10.0	26.7	32.3	0.101

Note: AH—arterial hypertension; TTH—tension-type headache.

**Table 2:** Allele frequencies and genotype distribution of rs3782218 of the *NOS1* gene.

Alleles, Genotypes	TTH and AH	AH	Control	*p*-Value
C	44 (73.3%)	55 (91.7%)	61 (98.4%)	0.000065 *, 0.08 **
T	16 (26.7%)	5 (8.3%)	1 (1.6%)	
CC	16 (53.3%)	25 (83.3%)	30 (96.8%)	0.000419 *, 0.078 **
CT	12 (40.0%)	5 (16.7%)	1 (3.2%)	
TT	2 (6.7%)	0 (0%)	0 (0%)	N/A *^,^**

Note: AH—arterial hypertension; TTH—tension-type headache; * *p*_1_-value—TTH and AH compared control; ** *p*_2_-value—AH compared control.

**Table 3:** Odds Ratio of tension-type headache and arterial hypertension overlap syndrome depending on minor or major alleles and genotypes of rs3782218 of the *NOS1* gene.

Alleles, Genotypes	χ^2^	*p*-Value	OR	95% CI
**TTH and AH vs. Control**				
C	15.959	0.000065	0.05	0.01–0.35
T			22.18	2.84–173.54
CC	15.556	0.000419	0.04	0.01–0.32
CT			20.0	2.4–166.97
TT			-	-
**AH vs. Control**				
C	2.945	0.086	0.18	0.02–1.59
T			5.55	0.63–48.95
CC	3.1	0.078	0.17	0.02–1.52
CT			6.0	0.66–54.79
TT			-	-
**TTH and AH vs. AH**				
C	6.984	0.008	0.25	0.09–0.74
T			4.0	1.36–11.77
CC	6.858	0.032	0.23	0.07–0.76
CT			3.33	0.99–11.14
TT			-	-

Note: AH—arterial hypertension; CI—confidential interval; OR—odds ratio; TTH—tension-type headache.

## Data Availability

Not applicable.
